# The inhibitory mechanism of Hal3 on the yeast Ppz1 phosphatase: A mutagenesis analysis

**DOI:** 10.1038/s41598-017-09360-5

**Published:** 2017-08-18

**Authors:** Cristina Molero, Carlos Casado, Joaquín Ariño

**Affiliations:** 1 Departament de Bioquímica i Biologia Molecular and Institut de Biotecnologia i Biomedicina, Universitat Autònoma de Barcelona, Bellaterra, 08193 Barcelona Spain; 2 Evolva Biotech A/S, Copenhagen, Denmark

## Abstract

The Ser/Thr protein phosphatase (PPase) Ppz1 is an enzyme related to the ubiquitous type-1 PPases (PP1c) but found only in fungi. It is regulated by an inhibitory subunit, Hal3, which binds to its catalytic domain. Overexpression of Ppz1 is highly toxic for yeast cells, so its de-regulation has been proposed as a target for novel antifungal therapies. While modulation of PP1c by its many regulatory subunits has been extensively characterized, the manner by which Hal3 controls Ppz1 remains unknown. We have used error-prone PCR mutagenesis to construct a library of Ppz1 variants and developed a functional assay to identify mutations affecting the binding or/and the inhibitory capacity of Hal3. We have characterized diverse Ppz1 mutated versions *in vivo* and *in vitro* and found that, although they were clearly refractory to Hal3 inhibition, none of them exhibited significant reduction in Hal3 binding. Mapping the mutations strengthened the notion that Hal3 does not interact with Ppz1 through its RVxF-like motif (found in most PP1c regulators). In contrast, the most relevant mutations mapped to a conserved α-helix region used by mammalian Inhibitor-2 to regulate PP1c. Therefore, modulation of PP1c and Ppz1 by their subunits likely differs, but could share some structural features.

## Introduction

Protein phosphorylation is a widespread mechanism for controlling cellular processes. The phosphorylation state of a given protein results from the opposing activities of protein kinases and protein phosphatases (PPases). Yeast cells contain different kinds of PPases that are also found in other eukaryotic organisms, including humans, while they also contain fungal-specific enzymes, such as the Ppz1 Ser/Thr protein phosphatase. Ppz1 is composed of a catalytic carboxyl-terminal domain, which is approximately 60% identical to the ubiquitous catalytic subunit of protein phosphatase 1 (PP1c), and a largely unstructured NH_2_-terminal extension^1^. In budding yeast, Ppz1 is involved in the regulation of monovalent ion homeostasis, by inhibiting the expression of the Na^+^/K^+^-ATPase Ena1^2, 3^ and the transport of potassium through the Trk1/Trk2 high-affinity transporters^4, 5^. Consequently, *ppz1* deletion mutants are tolerant to toxic Na^+^ and Li^+^ cations^2^. These regulatory effects impact on diverse cellular functions, such as cell wall integrity and osmotic stability^4, 6, 7^, translational efficiency^8^, and progress through the cell cycle^9^. It is worth noting that recent work has identified Ppz1 as one of the most toxic proteins when overexpressed in budding yeast^10^, although the molecular basis for this effect is unknown.

Ppz1 is regulated *in vivo* by Hal3/Sis2, a protein originally identified as a high-copy suppressor of the *sit4* cell cycle-related growth defect^11^ and by its capacity to confer halotolerance^12^. Hal3 binds to the carboxyl-terminal catalytic domain of Ppz1 and strongly inhibits its phosphatase activity, thus modulating its diverse physiological functions^13^. For that reason, cells overexpressing Hal3 are salt-tolerant, whereas a *hal3* strain is hypersensitive to sodium and lithium cations. Similarly, high-copy expression of Hal3 exacerbates the lytic phenotype of a Slt2 MAP kinase mutant, whereas, in contrast, lack of *HAL3* improves growth of this strain^13^. The *S. cerevisiae* genome contains a paralog of Hal3, named Vhs3, which also inhibits Ppz1 *in vitro*, although its role regulating the phosphatase *in vivo* is far less important, probably due to lower expression levels^14^. The ability of Hal3-related proteins to bind to and inhibit Ppz1 phosphatases has been demonstrated in the past for several, phylogenetically distant fungal species, suggesting that this is a conserved mechanism^15–18^.

This relatively simple scenario is complicated by the fact that Hal3 (and Vhs3) are moonlighting proteins that, in addition to regulating Ppz1, are components of an unusual heterotrimeric PPC decarboxylase (PPCDC) enzyme that is crucial for Coenzyme A biosynthesis in *S. cerevisiae* and other related fungi^16, 17, 19–21^. The functional heterotrimer requires Cab3, a Hal3-related protein that provides a catalytic Cys residue absent in Hal3 or Vhs3. Earlier work identified a number of residues in the conserved domain of *S. cerevisiae* Hal3 that are necessary for binding and/or inhibition of Ppz1, and revealed that these residues were unrelated to those required for PPCDC enzymatic function^22^.

The fact that Ppz1 is found only in fungi, including pathogenic ones, such as *Candida albicans* ^15, 23^ or *Aspergillus fumigatus* ^24^ and that increased Ppz1 function has a detrimental effect on yeast growth^9, 10, 13^ suggest that this enzyme may represent an interesting target for the development of antifungal therapies. However, because of the relative similarity between the catalytic domain of Ppz1 and that of the ubiquitous type 1 enzymes, it might be argued that specificity could be an important issue when interfering with Ppz1 function *in vivo*. In fact, when this work was started, there was evidence suggesting that diverse regulatory subunits of Glc7, the essential budding yeast type-1 phosphatase^25, 26^ could bind to Ppz1, and that at least one of them, Glc8, might regulate Ppz1-related functions at least partially^27^. However, other evidence pointed towards the possibility of significant regulatory differences between PP1c and Ppz1 enzymes. For instance, Ppz1 was shown to be less sensitive than rabbit PP1c to mammalian inhibitor-2^28^. In addition, the Glc7 regulatory subunit Ypi1 effectively binds to Ppz1, but barely inhibits its phosphatase activity *in vitro* nor it is able to mimic Ppz1-related Hal3 functions *in vivo* ^29^.

Interestingly, Hal3 does not appear structurally similar to known PP1c phosphatase inhibitors, with the exception of a KLHVLF sequence starting at residue 263, which resemble a relatively conserved sequence found in many PP1c regulatory subunits, known as the RVxF motif^30^. However, Hal3 does not bind to or inhibit the yeast PP1c *in vitro* ^13, 29^, indicating a strong specificity towards Ppz1. Moreover, mutation of the RVxF-like motif did not alter the *in vivo* or *in vitro* properties of Hal3^22^, suggesting that the mechanism by which Hal3 interacts and inhibits Ppz1 might differ from those found for the regulatory subunits of mammalian PP1c. On the basis of this evidence, we decided to study the mechanisms that allow the specific inhibition of Ppz1 by Hal3. To this end, we created a library of C-terminally targeted mutated versions of Ppz1 and developed a functional *in vivo* screen to search for deregulated enzymes. The results of this screen, presented here, confirm the notion that Hal3 inhibits Ppz1 in a way that differs from known regulators of mammalian PP1c enzymes, although it might retain some common structural features.

## Results

### Screen of a random-mutagenesis library for deregulated versions of Ppz1

The rationale of the screen was based on the previous observation that the absence of Hal3, the Ppz1 negative regulator, improves tolerance to caffeine in a cell-wall sensitive strain which lacks the Slt2 MAP kinase. Therefore, we hypothesized that a strain expressing a version of Ppz1 unable to bind to Hal3, or to be inhibited by it, could mimic the behaviour of the *hal3* mutation in a *slt2* background. Both centromeric and 2-micron vectors were tested, and finally the library was generated in the pRS316 centromeric plasmid to avoid the negative growth effect caused by high-copy number expression of Ppz1^13^. This library, carrying copies of Ppz1 randomly mutagenized between amino acids 351 and 59 nt downstream the stop codon, was transformed into strain JC010 (*slt2Δ*) and plated on synthetic medium including 4 mM caffeine. Under these conditions, *slt2* cells carrying a normal copy of Ppz1 cannot grow. Clones yielding macroscopic colonies after 3 days were selected, and their caffeine-tolerant phenotype was assessed by drop tests on plates containing 3, 4 or 5 mM caffeine. A total number of 36 clones were selected and the relevant Ppz1-encoding region sequenced. As shown in Supplementary File 1, several clones contained more than one change affecting the encoded amino acid. However, several clones included a single amino acid change and this subset was selected for further analysis (Table 1).

**Table 1:** Mutations affecting the catalytic domain of Ppz1 that result in single amino acid change.

**Clone**	**Nt position**	**Nt change**	**AA change**			**Ppz1 residue**	**Notes**
**136**	1165	GAA → AAA	Glu	→	Lys	389	
**99**	1282	TTA → GTA	Leu	→	Val	428	
**125** ^**(a)**^	1504	AAG → CAG	Lys	→	Gln	502	
**132**	1538	ACA → GCA	Thr	→	Ala	513	
**116**	1556	ATC → AGC	Ile	→	Ser	519	
**124**	1724	GAA → GGA	Glu	→	Gly	575	1
**151**	1735	AGT → GGT	Ser	→	Gly	579	
**97** ^**(b)**^	1889	GAA → GGA	Glu	→	Gly	630	
**149**	2053	ACA → CCA	Thr	→	Pro	685	

^(a)^Same than clone 126.

^(b)^Same than clone 141.

(1) Mutation also found in clones 139 and 143 (these clones carry an additional mutation at nt 1296 that do not change amino acid).

#### Phenotypic analysis of mutated alleles of *PPZ1*

**Figure 1:**
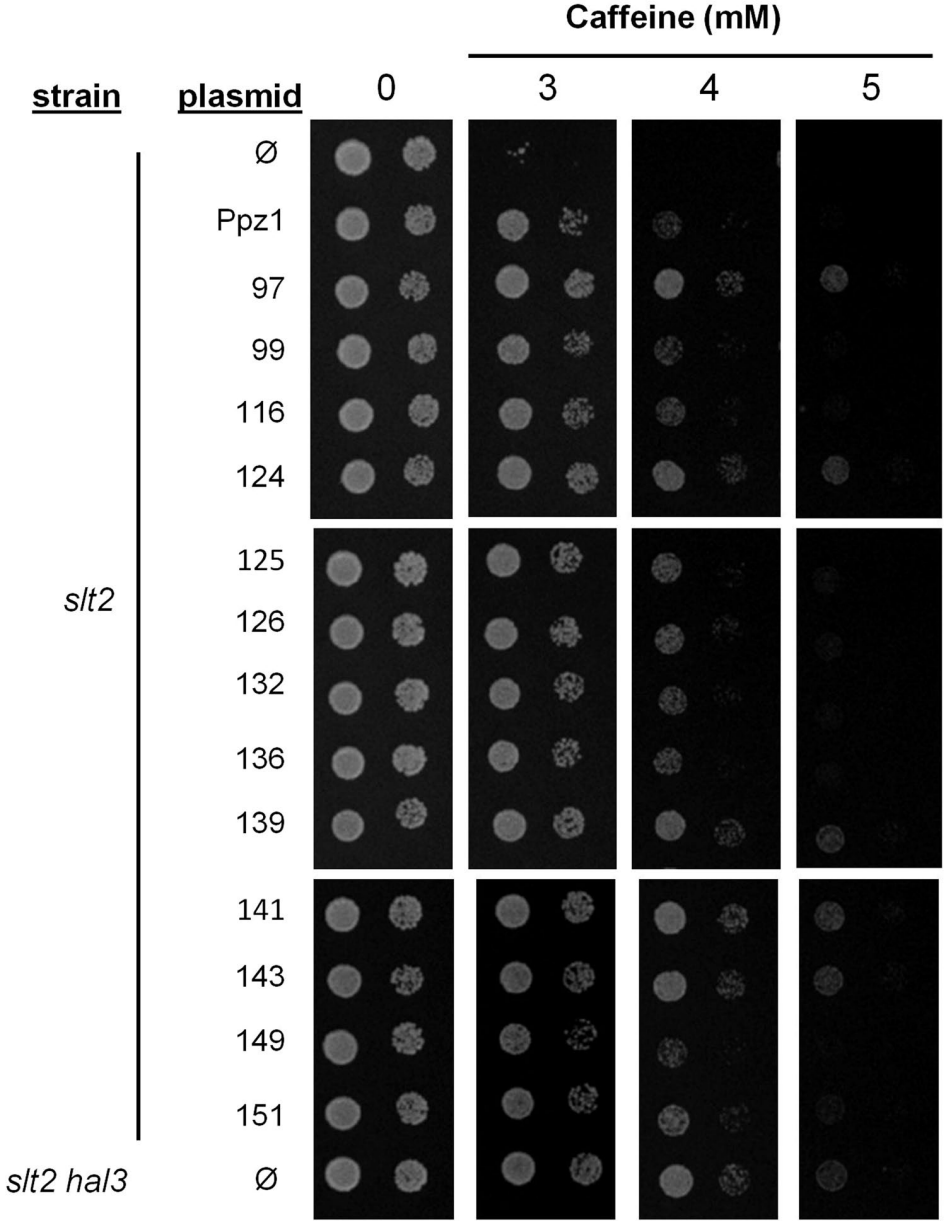
Growth on caffeine plates of selected Ppz1 clones carrying single amino acid mutations. The indicated strains were transformed with pRS316-based plasmids (centromeric, *URA3* marker) carrying the indicated versions of Ppz1. Cultures were spotted at OD_660_ = 0.05 and at 1/10 dilution, and plates were grown for 72 h. Ø, empty plasmid.

The growth of selected clones carrying a single amino acid mutation was examined on caffeine plates and compared with that of the *slt2* strain transformed with the native *PPZ1* gene and the *slt2 hal3* strain (which would mimic a largely derepressed Ppz1 activity). As shown in Fig. 1, clone 97 (and its equivalent 141) and clone 124 (and its equivalent 139 and 143) exhibited a rather strong phenotype, with a tolerance to caffeine higher than that of native Ppz1 and similar to that of the *slt2 hal3* strain. These clones harbor mutations of Glu to Gly at residues 630 and 575, respectively (Table 1). Clones 116 (Ile^519^ to Ser), 125 (Lys^502^ to Gln), 132 (Thr^513^ to Ala), 136 (Glu^389^ to Lys), and 151 (Ser^579^ to Gly) also showed higher tolerance than cells carrying native Ppz1, although the phenotype was less apparent. The behaviour of the different clones was also tested in liquid cultures, as documented in Supplementary File 2, and the time required to reach OD_600_ = 0.2 was calculated as described in Methods. These values confirmed that clones 97 and 124 exhibited the strongest phenotype (9.81 ± 0.09 and 10.44 ± 0.10 h, respectively) compared to cells carrying native Ppz1 (12.75 ± 0.09 h), and approaching that of *slt2 hal3* cells (8.92 ± 0.05 h).

**Figure 2:**
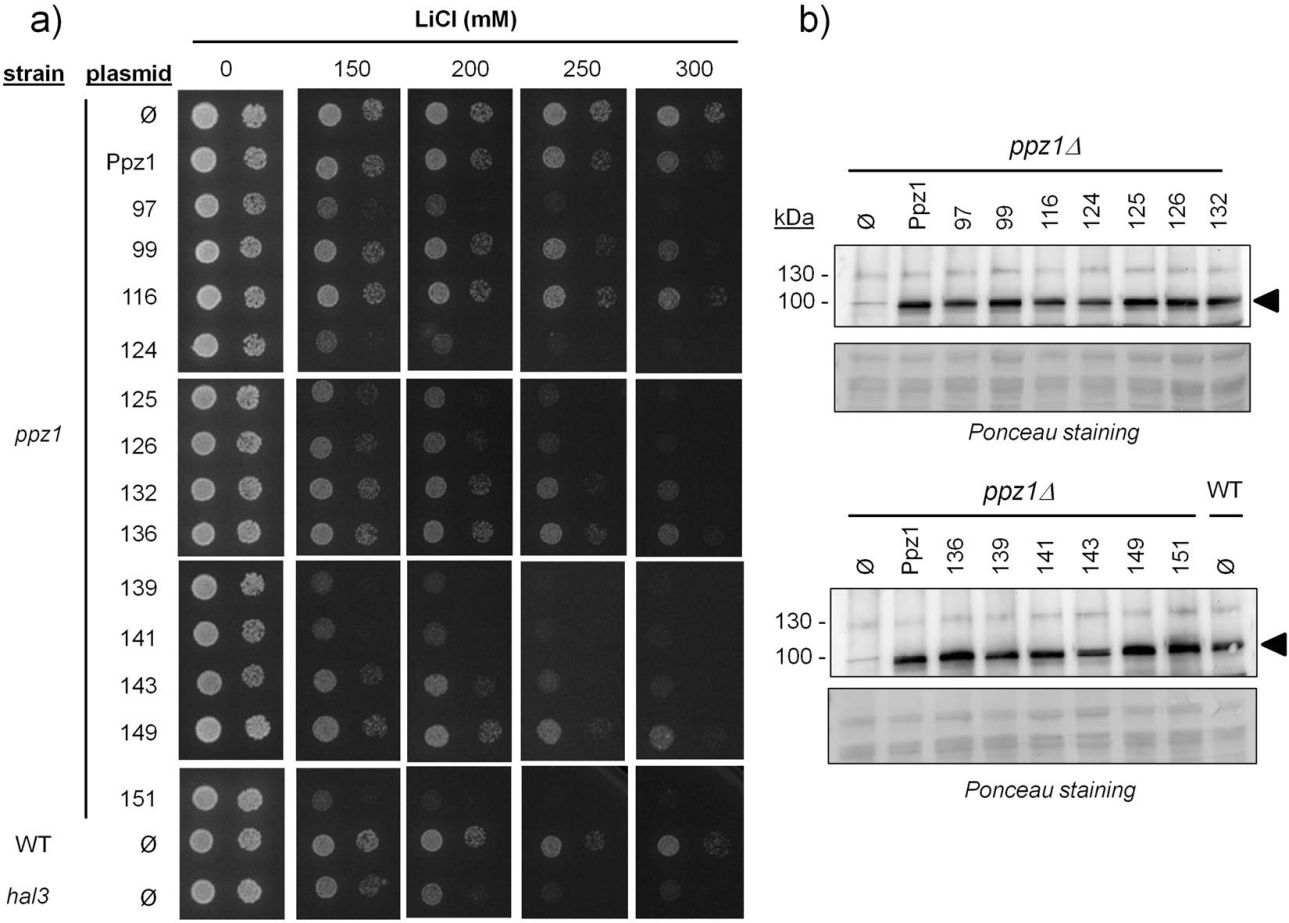
Tolerance to Li^+^ cations conferred by the diverse variants of the Ppz1 phosphatase. (**a**) The indicated strains were transformed with the diverse versions of Ppz1, and cultures were spotted as in Fig. 1 and grown for 72 h in the presence of various concentrations of LiCl. (**b**) Protein extracts (40 μg) from the indicated strains were subjected to electrophoresis on 10% polyacrylamide-SDS gels. Proteins were transferred to membranes and probed with a polyclonal anti-GST-Ppz1 antibody. Ø, empty plasmid; WT, wild type.

The activity of Ppz1 influences tolerance to toxic monovalent cations, such as Li^+^ and Na^+^. Thus, lack of Ppz1 increases tolerance to Li^+^, whereas increased Ppz1 activity, such as found in *hal3* mutants, decreases tolerance to this cation below the level of wild type cells. Thus, we tested the effect of expression of the diverse Ppz1 variants identified in our screen in cells exposed to high concentrations of Li^+^ cations. As shown in Fig. 2a, clones 97 and 124 showed again the strongest phenotype, similar to that of the *hal3* mutant. Clone 151 also displayed remarkable sensitivity to this cation, whereas clones 125 and 132 were nearly as sensitive as the *hal3* strain.

We then determined whether the different phenotypes observed could be the result of variations in the expression levels of the different Ppz1 variants. To this end, we prepared protein extracts from cultures of the different clones and carried out immunoblot analyses using a polyclonal antibody developed against recombinant Ppz1^1^. As shown in Fig. 2b, all variants of Ppz1 were expressed at fairly equal levels. Integration of the signals indicated that the possible differences were not higher than 20% with respect the mean expression value and that there was no correlation between the relative abundance of a given variant and its phenotypic effects (for instance, clone 124 showed the lowest expression value, but caused very strong phenotypes). In addition, the expression of the diverse variants did not differ from that of native Ppz1 expressed from its chromosomal locus. Therefore, the different cellular properties conferred by expression of the specific variants of Ppz1 are not likely to be due to variations in the amount of the phosphatase, but derived from the intrinsic properties of these variants. Interestingly, when the constructs 97 and 124 were introduced into a *slt2 hal3* strain, we did not observe any different in the tolerance to caffeine conferred by these variants in comparison with that of the wild type phosphatase (Supplementary File 3). Similarly, mutation of *HAL3* in the *ppz1* background did not allow distinguishing wild type from mutated variants when tolerance to lithium ions was tested (Supplementary File 3). These results strongly support the notion that the impact caused in the cell by expression of these Ppz1 variants is caused by the inability of these versions to be inhibited by Hal3.

During the course of the phenotypic characterization, we noticed a tendency to aggregate when certain Ppz1 variants were expressed in cells carrying the *slt2* mutation. This was interesting, because previous evidence linked the appearance of a flocculent phenotype to the hyperactivation of Ppz1^14, 31^. Therefore, we set up a quantitative flocculation test for the strains expressing the different Ppz1 versions. As shown in Supplementary File 4, expression of Ppz1 in the *slt2* background has no effect on flocculation, whereas additional deletion of *HAL3* increased the flocculation index by two-fold. Interestingly, *slt2* cells expressing Ppz1 variants 97 and 124 displayed a significantly increased flocculation index, further confirming that these two mutations greatly affected Ppz1 function *in vivo*. Cells expressing variant 141 (equivalent to 97), as well as variants 139 and 143 (equivalent to 124) also displayed an enhanced tendency to flocculate (not shown).

#### Determination of the capacity Hal3 to inhibit the Ppz1 variants

As described above, the aim of our screen was to identify Ppz1 residues important for binding to or inhibition by the negative regulator Hal3. However, it could be also argued that a similar effect could be achieved if a given mutation would enhance the activity of the phosphatase in a way that normal cellular levels of Hal3 would be unable of properly down-regulate Ppz1 activity. To evaluate this possibility, we expressed the different Ppz1 variants in *E. coli* as GST fusions, affinity-purified the proteins and, upon removal of the GST tag, tested their phosphatase activity. As observed in Supplementary File 5, the mutations did not greatly alter the specific activity of Ppz1 and, with the exception of clones 116, 124 and 151, the changes resulted in a phosphatase slightly less active than the native protein. These differences could be due, at least in part, to the difficulty to accurately determine the amount of purified recombinant Ppz1 (see Methods and Supplementary File 5) and, consequently, they might not reflect actual relevant differences. Therefore, it is unlikely that the observed phenotypic effects are derived from the expression of intrinsically hyperactive forms of the phosphatase.

**Figure 3:**
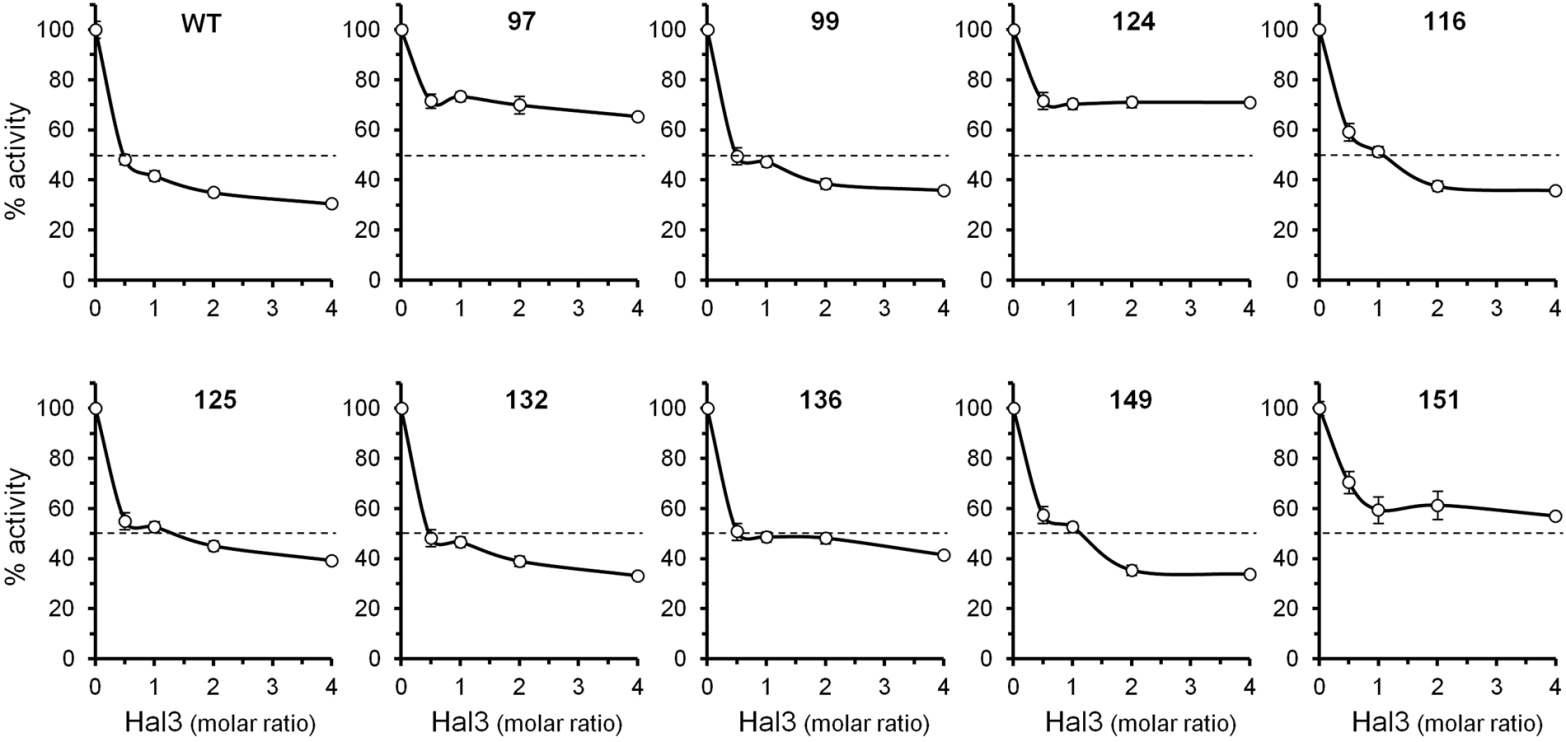
Ppz1 variants show differences in the pattern of *in vitro* inhibition by Hal3. *p*-nitrophenyl phosphate dephosphorylation assays were carried out as described under Methods. Six-hundred ng of the different affinity-purified Ppz1 variants were pre-incubated for 5 min with increasing amount of Hal3 prior starting the assay. Values are means ± S.E. for 4 to 12 different assays, and are expressed as the percentage of phosphatase activity relative to control without inhibitor. The dashed line at 50% activity is included to facilitate comparisons between panels.

We then tested the ability of Hal3 to inhibit the different versions of Ppz1 by incubating stoichiometric amounts of the inhibitor with the phosphatase prior to initiating the assay. As shown in Fig. 3, the Ppz1 versions derived from clones 97 and 124 were clearly refractory to inhibition by Hal3. The behaviour of Ppz1 from clone 97 closely corresponded to that observed for the phosphatase variant derived from clone 141, which harbours the same amino acid change. Similarly, the inhibition profiles of clones 139 and 143 were equivalent to that of clone 124. This was in agreement with the observation that these clones displayed the strongest phenotypic effects (see Figs 1 and 2). A similar behaviour was observed when these variants were incubated with Vhs3 (not shown), which inhibits Ppz1 *in vitro* with an efficiency similar to that of Hal3^14^. Ppz1 proteins from clones 151, 136 and, to some extent, from clone 125 (and its equivalent 126, not shown), were less sensitive to Hal3 inhibition than the native phosphatase. It must be noted that the inability of Hal3 to completely inhibit wild type full length Ppz1 is not surprising, since it is known that the N-terminal half of the phosphatase hinders binding of the inhibitor^13, 14, 16, 17^.

#### Ppz1 variants retain the ability to bind Hal3

**Figure 4:**
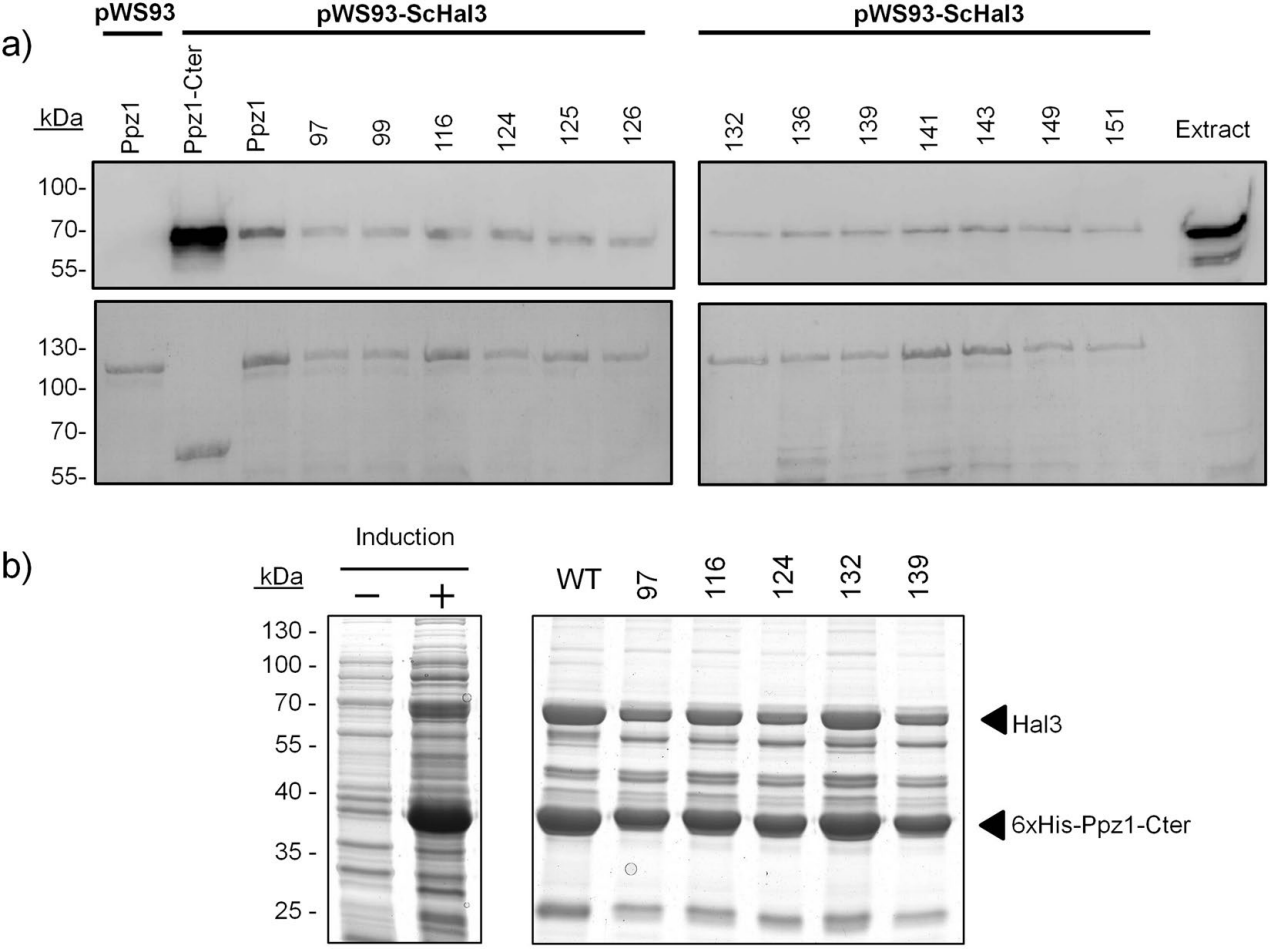
Interaction of Hal3 with the different Ppz1 variants. (**a**) Equal amounts of the indicated versions of GST-Ppz1 were immobilized on glutathione beads and used as an affinity system to recover plasmid-borne HA-tagged Hal3 from extracts of strain IM021 (*ppz1 hal3*). Beads were washed and processed for SDS-PAGE (8% gels) and immunoblotting using anti-HA antibodies as described in Methods. The lower panel corresponds to the Ponceau-stained membranes, to reveal the amount of Ppz1. (**b**) Co-expression and co-purification of Ppz1 variants and Hal3 in *E. coli*. Several 6xHis-tagged Ppz1-Cter versions carrying selected mutations were co-expressed together with untagged Hal3 in a polycistronic vector under the T7 promoter. The left panel shows bacterial extracts corresponding to the wild type Ppz1 as an example (−, non-induced; +, induced). The 6x-His tagged Ppz1-Cter proteins were purified by Ni-NTA agarose affinity chromatography and the protein complex subsequently eluted with buffer containing 500 mM imidazole. Samples (15 μl) were analyzed by 10% SDS-PAGE and proteins revealed with BlueSafe protein stain.

The observation that certain Ppz1 variants were not inhibited by Hal3 could be explained if the mutation in Ppz1 would abolish the ability of Hal3 to bind to the phosphatase. To test this possibility we used two different strategies. First, employed the recombinant versions of the GST-Ppz1 variants and used them as an affinity system to recover HA-tagged Hal3 from a yeast protein extract. The results of this experiment, shown in Fig. 4a, indicate that all tested Ppz1 variants interacted with quite similar efficiency. Quantification of the Hal3/Ppz1 signal ratio from 3 independent experiments yielded values ranging from 0.79 ± 0.09 (clone 116) to 1.22 ± 0.22 (clone 136), with a ratio for native Ppz1 of 1.14 ± 0.14. To confirm these findings we carried out a second approach based on the co-expression of a selection of the different His-tagged catalytic Ppz1 domains, together with Hal3, using a polycistronic vector. In this way, after affinity purification of Ppz1 using a Ni^+^-based resin, the untagged Hal3 is recovered only if it binds efficiently to Ppz1. The results of this approach are presented in Fig. 4b and confirm that Hal3 can be recovered in roughly the same amounts in all cases. Therefore, it can be assumed that the incapacity of Hal3 to inhibit Ppz1 versions such as 97 or 124 is related to the intrinsic inhibitory mechanism and not to substantial loss of binding to the phosphatase.

#### Characterization of additional *PPZ1* alleles

**Figure 5:**
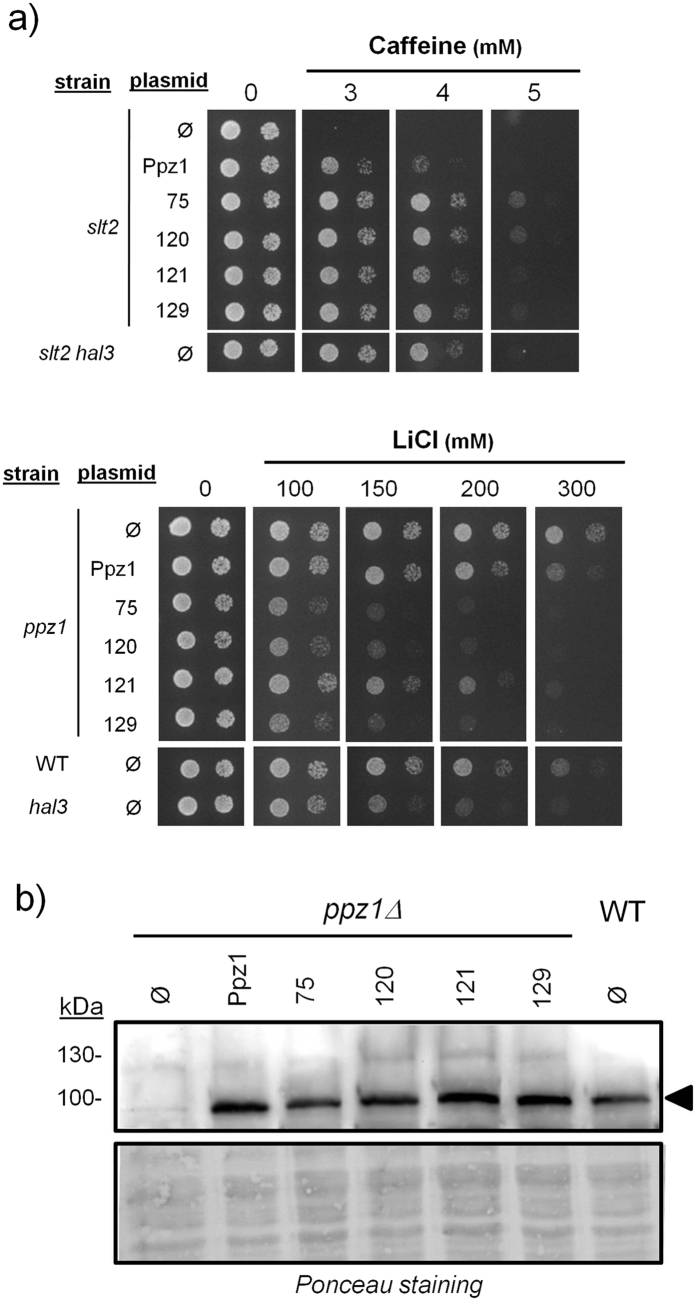
Phenotypic analysis and expression levels of selected Ppz1 versions carrying two amino acid changes. (**a**) Yeast strains were transformed with the diverse versions of Ppz1 and cultures spotted in the presence of various concentrations of caffeine or LiCl, as in Figs 1 and 2. Pictures were taken after 72 h. (**b**) Protein extracts (40 μg) were analyzed as described in Fig. 2b. Ø, empty plasmid; WT, wild type.

As documented in Supplementary File 1, our screen revealed numerous alleles carrying more than one amino acid changes. Among those, we selected four of them for further analysis (Table 2). Clones 75 and 120 were selected because they showed a strong phenotype in the initial screen and shared a common modification, the exchange of Val^605^ to Ala (the mutations in clone 120 were also present in clones 85, 100 and 140, see Supplementary File 1). Clone 121 showed the change of Phe^436^ and Phe^631^ to Leu, and was selected because Phe^631^ is the residue following the single Glu^630^ to Gly change found in clone 97 (identical mutations were found in clones 138, 153 and 168). Similarly, clone 129 carries the mutations Glu^365^ to Gly and Asn^574^ to Ser, of which the change affecting position 574 precedes the single Glu to Gly mutation found in clone 124. All four clones were evaluated for tolerance to caffeine in the *slt2* background and to Li^+^ cations in the *ppz1* background. As deduced from Fig. 5a and b, all clones exhibited a rather strong phenotype compared with that of cells carrying the wild type phosphatase. Tolerance to caffeine was also confirmed by continuous monitoring of growth in liquid culture (Supplementary File 6). In addition, when variants 120 and 121 were introduced in the *slt2 hal3* or in the *ppz1 hal3* backgrounds, and the resulting strains tested, respectively, for caffeine and LiCl tolerance, these phenotypic differences were abolished (Supplementary File 3). All four variants also increased the ability of *slt2* cells to flocculate (Supplementary File 4). These phenotypic effects were not due to differences in the amount of expressed Ppz1, as the protein levels of the different Ppz1 versions did not significantly differ from those of the native enzyme (Fig. 5b).

**Table 2:** Mutations affecting the catalytic domain of Ppz1 that result in changes in two amino acids.

**Clone**	**Nt position**	**Nt change**	**AA change**			**Ppz1residue**	**Notes**
**75** ^**(a)**^	1331	TTC → TCC	Phe	→	Ser	444	
	1814	GTT → GCT	Val	→	Ala	605	1
**120** ^**(b)**^	1070	TTC → TCC	Phe	→	Ser	357	
	1814	GTT → GCT	Val	→	Ala	605	
**121** ^**(c)**^	1306	TTC → CTC	Phe	→	Leu	436	
	1893	TTT → TTG	Phe	→	Leu	631	2
**129**	1094	GAA → GGA	Glu	→	Gly	365	
	1721	AAC → AGC	Asn	→	Ser	574	3

^(a)^Identical to clone 144.

^(b)^Identical to clones 85, 100 and 140. These clones include also changes at nucleotides 1806 (GCT to GCC) and 1845 (GAT to GAC) that do not modify the encoded amino acid.

^(c)^Identical to clones 138, 153 and 168.

(1) Same mutation than clone 120 (also found in clones 85, 100, 140, and 144).

(2) Follows single amino acid mutation in clone 97.

(3) Precedes single amino acid mutation in clone 124.

**Figure 6:**
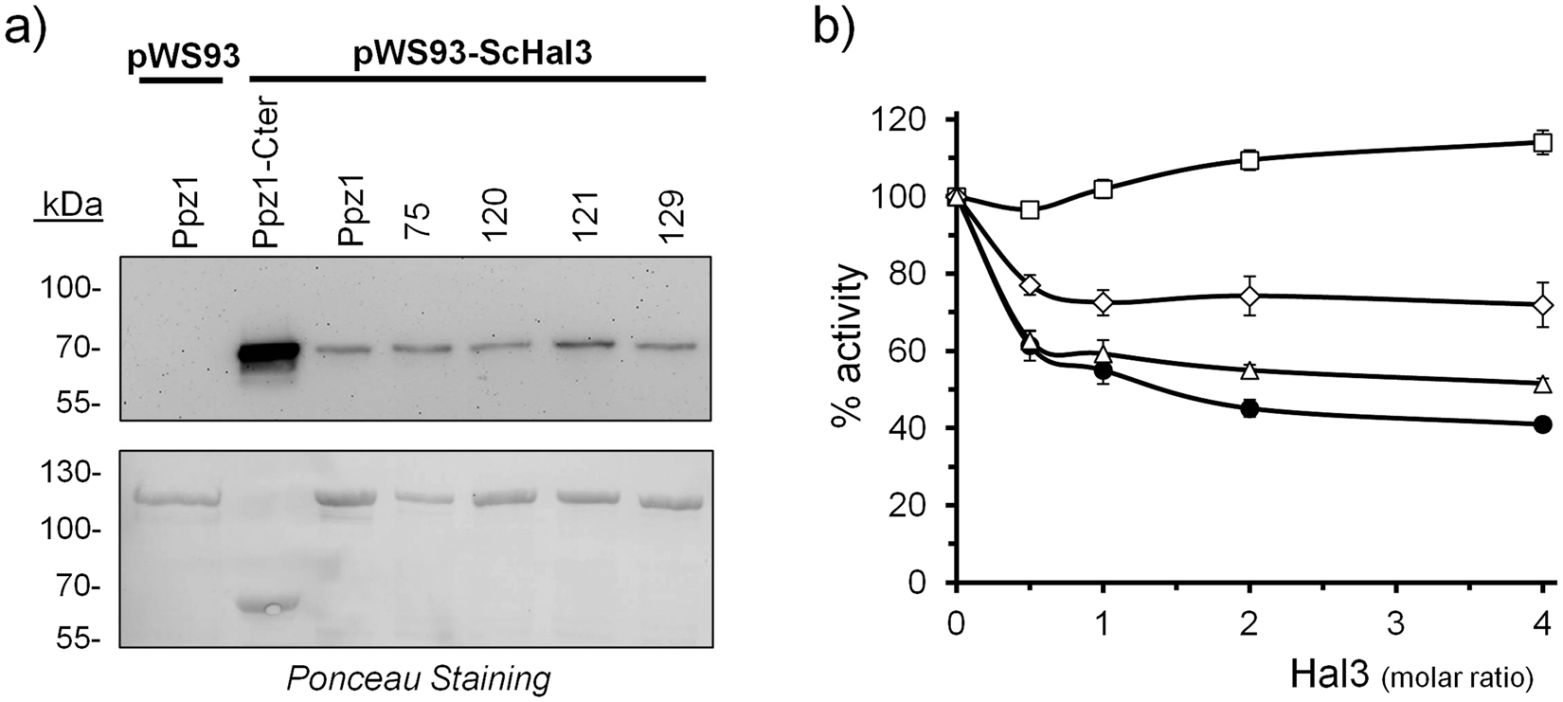
Hal3 binding capacity and inhibition profile of Ppz1 variants carrying two aminoacid mutations. (**a**) The indicated versions of GST-Ppz1 were immobilized on glutathione beads and processed as described in the legend of Fig. 4a. The upper panel reveals the amount of HA-tagged Hal3 bound to the phosphatase. The lower panel corresponds to the Ponceau-stained membranes, to show the amount of Ppz1. (**b**) Dephosphorylation assays were carried out as described in Fig. 3. Wild type Ppz1 (-●-); clone 120 (-□-); clone 121 (-◊-); clone 129 (-Δ-). Values are means ± S.E. for 6 to 10 different assays.

We then tested the ability of these Ppz1 variants to interact with Hal3. As shown in Fig. 6a, the different versions of Ppz1 expressed in *E. coli* as GST-fusion proteins and bound to glutathione-agarose beads were still able to bind Hal3 with similar efficiency. At this level we observed that expression in *E. coli* of clone 75 was clearly poorer than the wild type or the other variants. The ability of Hal3 to inhibit the Ppz1 versions carrying two amino acid changes was evaluated by removing the GST tag with the PreScission protease from the Ppz1 variants. As shown in Fig. 6b, Hal3 was completely unable to inhibit the Ppz1 corresponding to clone 120, whereas clone 121 was clearly refractory to inhibition compared with native Ppz1. The lack of inhibition observed for these versions fits well with the intensity of the phenotypes determined *in vivo*. In contrast, Hal3-mediated inhibition of the Ppz1 variant from clone 129 was only slightly less effective than that of native Ppz1. This was somewhat surprising, since the phenotypes observed upon expression of this clone in yeast were similar to those of clone 121. We were unable to perform inhibition experiments with the Ppz1 version from clone 75 since all attempts to release the phosphatase from the affinity matrix in a soluble and active form failed. Identical results were obtained using clone 144, which harbours the same two mutations. Clones 75 and 120 share a common mutation (Val^605^ to Ala), but differ in the second mutation. Since clone 120 was successfully expressed and showed full activity, we assume that the second mutation in clone 75 (Phe^444^ to Ser) could be responsible for the instability of the resulting protein when expressed in bacteria.

#### Mapping the mutations in Ppz1 and PP1c sequences

**Figure 7:**
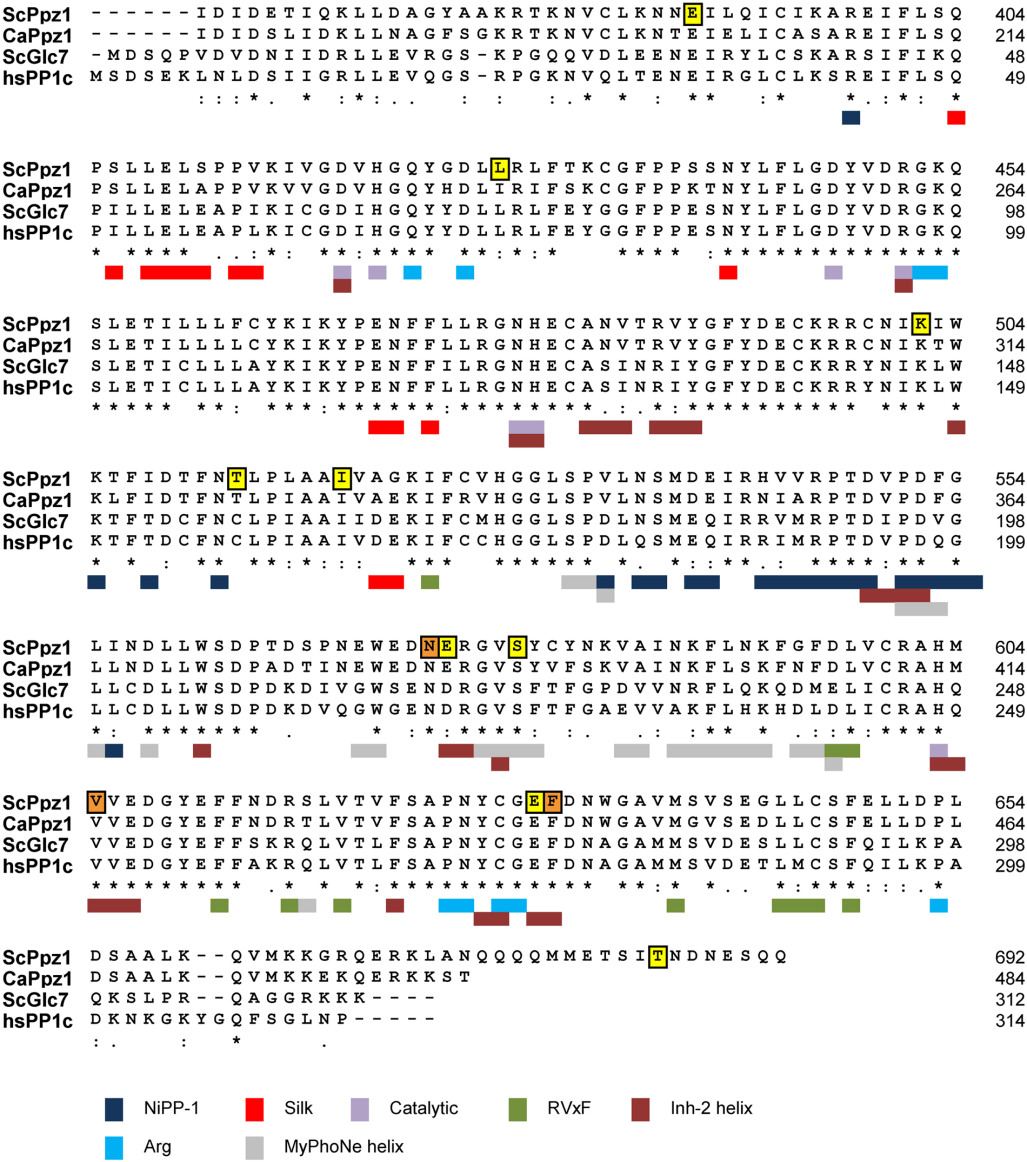
Alignment of the catalytic domains of Ppz1 from *S. cerevisiae* (ScPpz1) and *C. albicans* (CaPpz1), with the yeast (ScGlc7) and human (HsPP1c) catalytic subunit of type 1 protein phosphatase. The positions of the single mutations described in this work are denoted in yellow background, whereas the three relevant mutations found in clones 75, 121 and 129 are in orange. The positions of residues relevant for interaction with diverse motifs found in mammalian PP1c regulators (RVxF, SILK, NIPP-1, Arg, Inhibitor-2 helix), which are in some cases considerably conserved in Ppz1, or those forming the catalytic site, are indicated at the bottom of the sequences using a color code system. Numbering corresponds to the full length proteins. Inh-2, Inhibitor-2.

**Figure 8:**
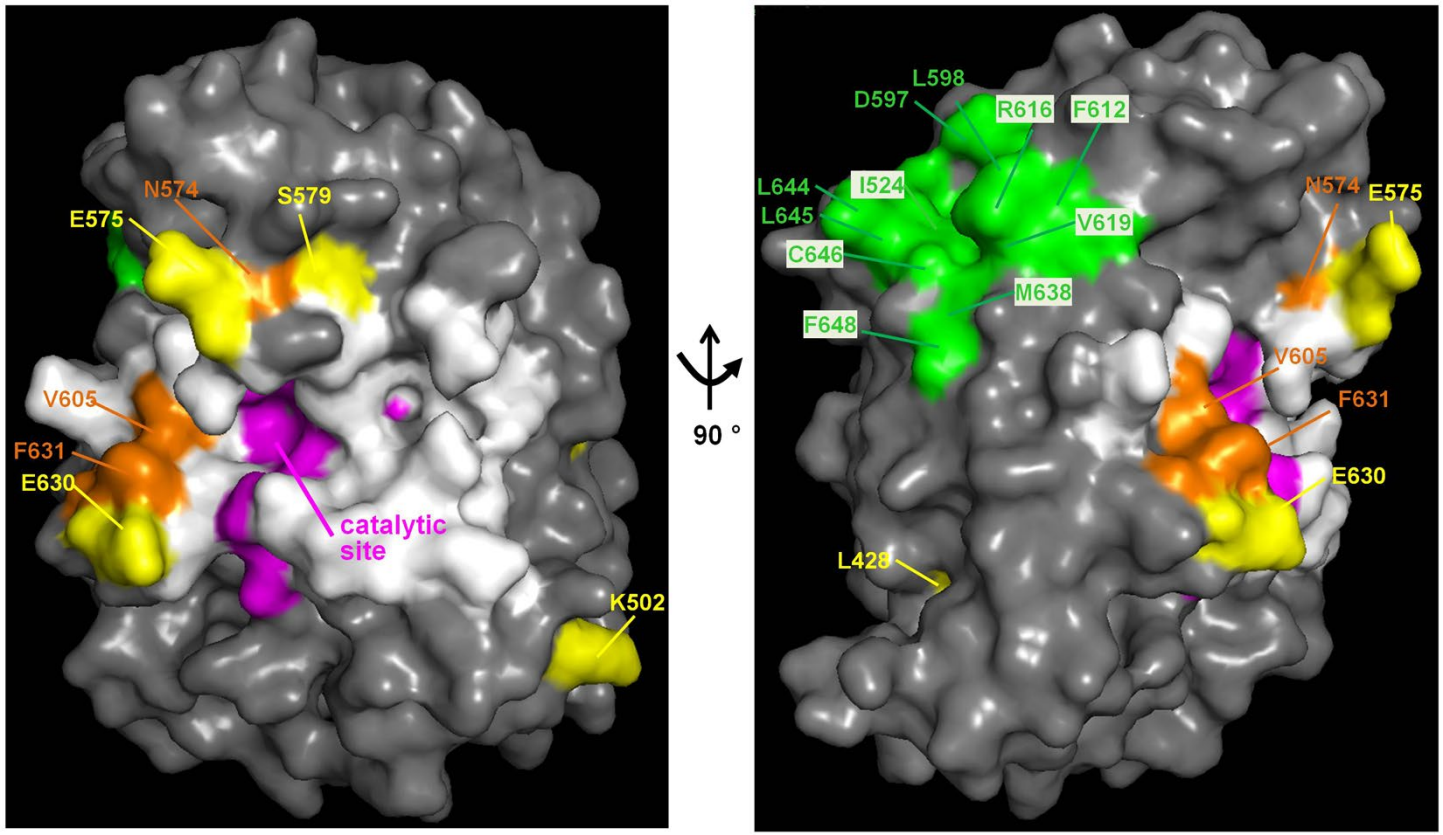
Mapping mutations and functional elements on a structural model of the C-terminal catalytic moiety of ScPpz1. The relevant mutations described in this work are highlighted in yellow and orange (as in Fig. 7). Residues involved in the catalytic mechanisms are pink, and the conserved hydrophobic groove, implicated in binding to RVxF-containing regulatory subunits, is denoted in green. Residues identical or similar to those involved in the interaction of mammalian PP1c with Inhibitor-2 α-helix described in the text are highlighted in white. Note that E^575^, V^605^, E^630^, and F^631^, as well as diverse residues in the catalytic site, although not depicted in white, are also involved in PP1c interaction with Inhibitor-2. The correspondence between the annotated residues and the clones recovered from the screen can be found in Tables 2 and 3.

The sequences corresponding to the C-terminal halves of *S. cerevisiae* and *C. albicans* were aligned with that of the entire *S. cerevisiae* (Glc7) and human PP1 catalytic (PP1c) subunits and the different mutations characterized in this work were highlighted in the ScPpz1 sequence (Fig. 7). Based on the work of Chen and coworkers^32^, we selected those residues that define the regulatory motifs in mammalian PP1c that are highly conserved in the fungal Ppz1 proteins, such as those required for interaction with the RVxF, Inhibitor-2 helix, Arg, NIPP1-helix, or SILK motifs present in regulatory subunits of PP1c, and annotated them in the alignment. Comparison of the selected residues with the mutations found in this work clearly shows that, as a rule, none of the mutations are within these regulatory binding motifs, in particular with the highly conserved RVxF-binding motif present in most PP1c regulatory subunits. Notably, the only exception were Glu^575^, Val^605^, Glu^630^, and Phe^631^, which corresponded to PP1c residues (Glu^575^ being an Asp residue in PP1c) known to interact with the Inhibitor-2 helix^33^. It must be noted that these are the Ppz1 mutations that cause the strongest phenotypes. To obtain a deeper insight into the relative position of these mutations in the *S. cerevisiae* Ppz1 protein we created a three-dimensional model of the budding yeast phosphatase C-terminal domain, taking advantage of the recently solved 3D structure of the catalytic domain of its *C. albicans* homolog. As shown in Fig. 8, virtually all of the functionally relevant mutations found in this work are located at the surface and on the same side of the phosphatase, which corresponds to the access to the catalytic site. This clustering of mutations fits well with a role for these residues being relevant in the modulation of the phosphatase activity by the Hal3 inhibitor.

## Discussion

Modulation of the functions of Ser/Thr phosphatases by means of their interaction with regulatory subunits is a recurrent scenario in the biology of the cell. In addition to the ubiquitous eukaryotic PP1c homolog Glc7, the budding yeast *S. cerevisiae* possesses three additional, structurally related PPases, Ppz1, Ppz2 and Ppq1/Sal6^34^. Functional studies have revealed a large number (>25) of regulatory subunits of Glc7^35^, whereas only two of them (Hal3 and Vhs3) have been identified for Ppz1 (and Ppz2) and, as far as we know, there is no evidence for Ppq1/Sal6 regulatory subunits. Glc7 provides essential functions to the yeast cell that are not disrupted by the presence of other fungus-specific phosphatases, which suggest that, for the most part, Glc7 regulation is separated from that of Ppz1. It is worth noting that, in contrast to the existing wealth of information about the mechanisms of regulation of mammalian PP1c (and also yeast Glc7), very little is known about how Hal3, the major regulator of Ppz1, exerts its function.

The majority (about 2/3) of PP1c (and Glc7) regulators are proteins containing the so-called RVxF motif^30^, which binds to a hydrophobic groove whose structure is strongly conserved in Ppz1 (see Figs 7 and 8). There are a number of observations supporting the notion that the RVxF-binding motif is also functionally conserved in Ppz1. For instance, *in vivo* interactions between Ppz1 and two Glc7 regulatory subunits displaying RVxF motifs, Glc8 and Ypi1, has been reported by 2-hybrid experiments^27^. Interaction between Ppz1 and Ypi1 was also documented by pull-down assays, and it was demonstrated that a W53A mutation in its ^48^RHNVRW^53^ sequence, which resembles the characteristic consensus PP1c binding motif, abolished binding to both the Glc7 and Ppz1 phosphatases^29^. In addition, both *S. cerevisiae* and *C. albicans* Ppz1 are sensitive to mammalian Inhibitor-2, a PP1c regulatory subunit that contains a ^44^RKLHY^148^ sequence that functionally replaces the RVxF motif^36^. Hal3 contains a ^263^KLHVLF^268^ sequence that also resembles a RVxF motif. However, it has been shown that mutation of H^265^ or F^268^ does not affect binding nor inhibitory capacity of Hal3 upon Ppz1^22^, suggesting that this likely RVxF motif is not relevant for the interaction with Ppz1. This scenario is in agreement with the fact that we did not recover any mutation affecting the hydrophobic groove involved in the binding to RVxF motifs in our screen. Therefore, Hal3 must interact with Ppz1 by means of other structural elements different from the RVxF motif. Sequence comparisons and recent experimental evidence gathered from studies of the *C. albicans* Ppz1 C-terminal domain^32^ indicate that diverse docking motifs found in PP1c, such as that of either PNUTS or spinophilin, are not likely to be relevant for yeast Ppz1. The observation that none of the Ppz1 versions tested in this work shows significant loss of binding capacity, whereas some of them are clearly refractory to Hal3 inhibition, might imply that Ppz1-Hal3 contacts are maintained by multiple interacting elements, still to be defined. In this regard, it is worth noting that these interacting determinants should differ from those of type-1 PPases, since it has been shown that Hal3 does not bind to Glc7 *in vitr*o^13, 29^.

As mentioned above, mutations strongly affecting Ppz1-related phenotypes *in vivo*, as well as the ability to be inhibited by Hal3 *in vitro*, are located at the surface and on the same side of the phosphatase, surrounding the access to the catalytic site (Fig. 8). This is interesting because it has been shown that residues 130–169 of inhibitor-2, which form the so-called binding site 3, lie along the acidic and hydrophobic substrate binding channel of PP1c^37^. This channel is strongly conserved in fungal Ppz1 proteins and includes several residues involved in the catalytic mechanism. We observe that most of the phenotypically relevant mutations found in this work, affecting Glu^575^, Val^605^, Glu^630^, and Phe^631^, alter residues which are required for PP1c to interact with inhibitor-2. It must be noted that the nature of our screen precludes the isolation of mutations altering catalytic residues (likely leading to loss of phosphatase activity). Therefore, it could be hypothesized that the inhibitory strategy of Hal3 on Ppz1 might mimic that of Inhibitor-2, despite the fact that both proteins exhibit almost no sequence similarity (it must be stressed that the overall sequence similarity of Inhibitor-2 and Glc8, its presumed yeast homolog, is also very low, <30%). Recently, Choy and coworkers^38^ postulated that an extended PP1c-interacting motif could be identified in diverse regulatory subunits. Such an extended motif would initiate with a more or less degenerate RVxF motif and would present the sequence -RVxF-n_(5-8)_-ΦΦ-n_(8-9)_-R-, where Φ are hydrophobic residues (commonly -VF-). It is remarkable that Hal3 presents a similar sequence -HVLF-n_(7)_-VF-n_(6)_KK-, where HVLF is the non-functional RVxF-like motif and the Arginine is replaced by another basic residue, Lysine. An equivalent sequence, -HVLF-n_(7)_-VF-n_(6)_-RK-, is also found in the second Ppz1 inhibitory subunit, Vhs3. In contrast, the equivalent sequence in Cab3, a Hal3-related protein that cannot inhibit Ppz1, is -HILI-n_(7)_-TI-n_(6)_-DK-, thus lacking both the ΦΦ and basic elements. It would be interesting to explore whether or not this structural feature has any relevance in Hal3.

In conclusion, the present work provides an initial clue to decipher the mechanism of inhibition of fungal Ppz1 by Hal3. It must be noted that Hal3 is a moonlighting protein, acting both as an inhibitor of Ppz1 and as a member of an essential heterotrimeric PPCDC enzyme. Accordingly, the core domain of *S. cerevisiae* Hal3, required for efficient interaction with Ppz1^20, 22^, is predicted to display a structured architecture, in contrast with most PP1c-interacting proteins, which are intrinsically disordered^39^. It is likely that the dual function of Hal3 has determined the evolutionary pace of the protein, thus developing an inhibitory mechanism that may present differences from what is currently known for PP1c regulatory subunits. In this regard, it is worth noting the important role of the N-terminal segment of Ppz1 (absent in Glc7), consisting of a Ser/Thr rich, basic sequence, likely unstructured, which was described long ago to interfere with the binding of Hal3 to the *S. cerevisiae* Ppz1 catalytic moiety^13^ and more recently, in the case of *C. albicans* Ppz1, to reduce the inhibitory capacity of mammalian inhibitor-2^32^. This type of mechanism would help to further insulate Ppz1 and Glc7 functions in yeast.

## Methods

### Yeast strains and growth conditions

*Saccharomyces cerevisiae* cells were grown, unless otherwise stated, at 28 °C in YPD medium (10 g/L yeast extract, 20 g/L peptone, and 20 g/L dextrose) or in complete minimal medium (CM) lacking the appropriate requirements when carrying plasmids for selection^40^. All yeast strains used in this work are listed in Table 3.

**Table 3:** Yeast strains used in this work.

**Strain**	**Relevant genotype**	**Source/Reference**
JA100	*MATa ura3*-52 *leu2*-3, 112 *his4 trp1*-1 *can*-1r	13
JC010	JA100 *slt2*::*LEU2*	47
AGS9	JA100 *ppz1*::*LEU2*	48
EDN75	JA100 *ppz1*:: *KANMX4*	8
JA104	JA100 *hal3*::*LEU2*	13
CCV186	JA100 *slt2*::*LEU2 hal3*::*KANMX4*	16
IM021	JA100 *ppz1*::*KANMX4 hal3*::*LEU2*	22

### Recombinant DNA techniques and plasmids

*Escherichia coli* DH5α strain was used as plasmid DNA host and was grown in LB medium at 37 °C supplemented with 50 μg/ml ampicillin when needed for plasmid selection. *E.coli* strain BL21 (DE3) RIL was used for heterologous protein expression as described below. Restriction reactions, DNA ligations, and other standard recombinant DNA techniques, including bacterial and yeast transformations were performed using standard methods.

The source for the *PPZ1* gene was plasmid YCp33-PPZ1, which contains a 2.7-kbp DNA genomic fragment cloned into BamHI/HindIII sites^41^. Plasmid pRS316-PPZ1 was constructed by digestion of YCp33-PPZ1 with BamHI/HindIII and cloning of the 2.7-kbp fragment into the same sites of plasmid pRS316^42^. The construction of plasmid YEplac195-PPZ1 was previously described^9^. The pGEX-6P1 (Amersham Biosciences) plasmid was used to express N-terminally GST tagged recombinant proteins in *E. coli*. Constructs expressing the entire *S. cerevisiae* Ppz1 phosphatase (pGEX6P1-Ppz1) and its C-terminal catalytic domain (lacking residues 1-344) (pGEX6P1-Ppz1^Cter^), as well as pGEX6P1-Hal3, coding for the entire Hal3, have been described previously^14^.

### Random PCR Mutagenesis and screen for Ppz1 deregulated versions

Random PCR mutagenesis was performed essentially as in Fromant *et al*.^43^ using MgCl_2_ at a final concentration of 5.5 mM. Plasmid pRS316-Ppz1 and oligonucleotides 5′-PPZ1_BsrGI (5′-TGTGCATATGTACATCGTTGAG-3′) and 3′-PPZ1_BspEI (5′-TAATGCAATCTTCCGGAAAC-3′) were used to amplify the 1.2 kbp fragment between the BsrGI and the BspEI sites corresponding to the catalytic domain of Ppz1 (the later located 59 nt downstream the stop codon). Four different reactions were made in which the forcing dNTP was at a concentration of 3.4 mM, whereas the other dNTPs were at 0.2 mM. The products of various independent PCR reactions were pooled, purified by phenol/chloroform/isoamyl alcohol, followed by ethanol precipitation, and digested with BsrGI and BspEI to clone in the same sites of the gapped plasmid pRS316-PPZ1. Ligation products were introduced into *E. coli* competent cells by electroporation^44^. Approximately 25,000 independent colonies were recovered and mixed.

Strain JC010 (*slt2*Δ) was transformed with the plasmid library (1 µg DNA/transformation). Approximately 36,000 transformants were plated in CM-uracil medium containing 4 mM caffeine (around 3,000 transformants/plate, as determined using control CM plates lacking uracil (CM-uracil)). Plates were incubated for 48–72 h at 28 °C. Clones able to generate macroscopic colonies under these conditions (usually 10–20/plate) were picked out and grown for an additional 3–6 h in sterile 96-well plates filled with CM-uracil medium. They were then spotted on CM-uracil plates and in the same plates containing 3 mM, 4 mM or 5 mM caffeine for initial characterization. Plasmids from the selected clones were then extracted, amplified in *E. coli*, and reintroduced into strain JC010 to reassess the phenotype as indicated above. The catalytic domain of Ppz1 from plasmids providing sustained tolerance to caffeine was subjected to sequencing in search of mutations producing a change in the amino acidic sequence of the protein.

### Yeast protein extraction and immunoblot analysis

Cells containing plasmids expressing the different mutated versions of Ppz1 were tested for integrity and expression levels of the Ppz1 protein. For this purpose, *S. cerevisiae* strain AGS9 (*ppz1Δ*) was transformed with the different constructs and cells were grown in 10 ml of synthetic minimal medium lacking uracil at 28 °C to A_660_ of 0.8–0.9. Protein extracts were prepared as described in^20^, and forty μg of total protein were resolved by SDS-PAGE. For immunobloting, proteins were transferred to Immobilon-P membranes (Millipore), and Ppz1 was immunodetected using anti-GST-Ppz1 polyclonal antibodies (1:250 dilution), followed by anti-rabbit peroxidase secondary antibodies (GE Healthcare) at a 1:20,000 dilution. The immunocomplexes were visualized using ECL Western blotting detection Kit (GE Healthcare).

### Expression of Ppz1 mutated versions in *E. coli*

The selected mutated versions of *PPZ1* were expressed in *E. coli* as follows. The BspEI/BsrGI fragment containing the specific mutations was removed from the pRS316-borned construct by digestion with the indicated enzymes, and the resulting 1.2 Kbp fragments cloned into the same sites of BspEI/BsrGI gapped pGEX6P1-Ppz1 plasmid. The different versions of Ppz1, as well as the Hal3 inhibitor, were expressed *E. coli* BL21 (DE3) RIL cells as GST-fusion proteins and purified essentially as previously described^14, 19, 29^ except that in all cases induction was performed using 0.1 mM isopropyl-1-thio-β-D-galactopyranoside, and cultures were grown at 20 °C overnight. Once bound to the glutathione-agarose affinity column, when required the recombinant proteins were treated overnight at 4 °C with PreScission protease (Amersham Biosciences) following the manufacturer’s indications (80 units/ml resin) to cleave the GST moiety. The eluted GST-free proteins were analyzed by SDS-PAGE followed by Coomassie Blue-staining (see Supplementary File 5). Due to the presence of lower molecular mass proteins the amount of each full-length Ppz1 variant was determined by scanning of the gel using Gel Analyzer software, integration of the Ppz1 band and comparison with different amounts of bovine serum albumin.

### Assay of protein phosphatase activity

The capacity of Hal3 or Vhs3 as inhibitors of the different mutated versions Ppz1 was analyzed using recombinant proteins. To this end, the Ppz1 phosphatase activity was measured using *p*-nitrophenylphosphate as substrate essentially as described previously^20, 29^ with the following modifications: 600 ng of Ppz1 phosphatase was used, the concentration of substrate was 10 mM, and the assay was carried out for 20 min at 30 °C. Each phosphatase version was incubated with different amounts of the inhibitor for 5 min at 30 °C, and the assay was started immediately by the addition of the substrate.

### Detection of protein-protein interactions

The ability of diverse Ppz1versions to bind Hal3 was assessed by two different approaches. From one side, *in vitro* binding assays were performed as follows. Aliquots of the glutathione-agarose beads containing 4 μg of GST-Ppz1, GST-Ppz1-Cter, and the diverse GST-Ppz1 mutated versions (∼50 μl), were mixed with protein extracts prepared from strain IM021 (*ppz1 hal3*) transformed with the multicopy plasmid pWS93 (negative control) and pWS93-Hal3, carrying a HA-tagged version of Hal3. The amounts of extracts used were 0.2 mg for cells expressing *S. cerevisiae* Hal3, and 2.0 mg for negative controls. Binding conditions and analysis were as in^20^. Samples (15 μl) were analyzed by SDS-PAGE and probed using anti-HA antibody (Covance). Membranes were subsequently stained with Ponceau Red to reveal the amount of GST-Ppz1 loaded. The relative amounts of Hal3 and Ppz1 were determined by integrating the immunoreactive Hal3 and the Ponceau-stained GST-Ppz1 signals with the Gel Analyzer software and calculating the Hal3/GST-Ppz1 ratio.

Alternatively, co-expression of Ppz1-Cter carrying selected mutations and Hal3 was carried out in *E. coli* using a polycistronic vector (T7 promoter), in a way that a 6xHis-tagged version of Ppz1-Cter is isolated by Ni-affinity chromatography and the co-expressed Hal3 protein is recovered on the basis of its capacity to interact with Ppz1-Cter. To this end, N-terminally 6xHis tagged Ppz1-Cter versions and Hal3 were cloned independently into pET His TEV LIC cloning vector (2B-T), a gift from Scott Gradia (Addgene plasmid # 29666), to provide the required ribosome binding sites, and then both ORFs were transferred to the multicloning site (cassettes 1 and 2) of the pET empty polycistronic destination vector (2E) (Addgene plasmid # 29775). Details of the construction steps will be published elsewhere. The polycistronic vector generated, named A2-Ppz1-Cter + Hal3, was used to transform DH5α competent cells. Positive colonies grown in LB agar plates plus 30 mg/ml Kanamycin were recovered and the inserts sequenced to verify the absence of unwanted mutations.

Expression was performed in chemically transformed *E.coli* BL21 (DE3) RIL cells grown in LB supplemented with 30 µg/ml kanamycin and 34 µg/ml chloramphenicol by an auto-induction method. For this, a preculture of 10 ml of LB supplemented with 1% glucose and the selection antibiotics was grown at 37 °C for 16–18 h at 230 rpm. This culture was used to inoculate 50 ml of auto-induction media (to be described elsewhere) supplemented with 100 µg/ml kanamycin to an OD_600_ of at least 0.05, and the culture was growth at 230 rpm and 37 °C to reach a density of OD_600_ = 0.6–1.2. At this point, temperature was decreased to 20 °C and growth continued for about 48 h. Cells were then collected by centrifugation at 6,500 xg for 20 min at 4 °C and pellets were resuspend in 8 ml of binding buffer (50 mM Tris-HCl pH 8, 100 mM NaCl, 10 mM imidazole) supplemented with 0.1% Triton X-100, 1 mM DTT, 1 mM PMSF and protease inhibitor cocktail (complete EDTA-free, Roche). The suspension was sonicated and then centrifuged at 4 °C (12,000 xg for 30 minutes) to remove cellular debris. The 6x-His tagged Ppz1-Cter protein was purified from the cell extract by gravity-flow chromatography using an Ni-NTA agarose matrix (Quiagen). After loading the lysate, the matrix was washed with binding buffer supplemented with 50 mM imidazole, and the protein complex subsequently eluted after incubation for one h with the same buffer containing 500 mM imidazole. Samples (15 μl) were analyzed by SDS-PAGE and stained with Coomassie Blue or BlueSafe (#MB15201, NzyTech).

### Phenotypic analysis of yeast cells

Tolerance to caffeine or LiCl of *S. cerevisiae* cells carrying the plasmids with the different Ppz1 mutated versions was evaluated by growth on plates (drop tests) as described previously^2^. Tolerance in liquid cultures was tested by monitoring growth rate of a starting culture (OD_600_ nm of 0.05) for 24 h in a Bioscreen C equipment (Labsystems). Measures were taken every 30 min. The calculation of the time needed to reach a given OD was calculated by solving the exponential equation fitted to the growth curve. Flocculation assays were performed as in^45^ except that after the addition of calcium chloride (that induces flocculation), tubes were shaken at 28 °C (230 strokes/min) for 5 min and left standing for 5 min. A sample of the supernatant was then taken, made 0.25 M EDTA, and the OD_600_ measured. The flocculation index is calculated as the ratio of the OD_600_ between the fully deflocculated cell suspension and the EDTA-treated supernatant.

For modelling studies the sequence comprising residues 361 to 692 of S. cerevisiae Ppz1 was submitted to the Swiss-Model Web Sever^46^. Models were built based on the target-template alignment using ProMod3 and the 3D-structure of *C. albicans* C-terminal Ppz1 protein (5jpe)^32^.

### Data availability

All data generated or analyzed during this study are included in this published article (and its Supplementary Information files). Protocols, DNA constructs, and other materials generated in this work will be made available without restrictions upon request to the corresponding author.

## Electronic supplementary material

Supplementary Table and figures
