# Supplementary material for: The inhibitory mechanism of Hal3 on the yeast Ppz1 phosphatase: A mutagenesis analysis
Source: Sci Rep. 2017 Aug 18;7:8819. doi: 10.1038/s41598-017-09360-5 (PMC5562863; doi:10.1038/s41598-017-09360-5)
Supplement: Supplementary file 1 — Supplementary Table and figures [file 41598_2017_9360_MOESM1_ESM.pdf]

The inhibitory mechanism of Hal3 on the yeast Ppz1 phosphatase: A mutagenesis analysis

Cristina Molero, Carlos Casado, Joaquín Ariño

Legends to Supplementary Files

Supplementary File 2. Growth curves of *slt2* cells carrying alleles of Ppz1 harboring single amino acid mutations. Strain JC010 (*slt2*) was transformed with the indicated pRS316-based Ppz1 variants . Cultures were inoculated at OD_{600} of 0.05 in liquid synthetic medium lacking uracil and supplemented with 4 mM caffeine. Growth was monitored up to 24 h at 28 °C by measuring OD_{600} every 30 min in a Bioscreen C equipment (Labsystems). pØ, empty plasmid; WT, wild type.

Supplementary File 3. Deletion of *HAL3* equalize the effect of expressing native Ppz1 and its variants. A) Strain CCV186 (*slt2 hal3*) was transformed with pRS316-based plasmids (centromeric, *URA3* marker) carrying the indicated versions of Ppz1 and cultures were spotted at OD_{660}=0.05 and at 1/10 dilution. B) Strains EDN75 (*ppz1*) and IM021 (*ppz1 hal3*) were transformed with the same plasmids and their tolerance to LiCl tested as above. Growth was recorded after 72 h of incubation. Ø, empty plasmid.

Supplementary File 4. Deregulated alleles of Ppz1 induce flocculation in a Slt2-deficient background. The indicated strains were transformed with the empty plasmid (Ø), with the wild type version of *PPZ1* (WT), or with the indicated *PPZ1* alleles carrying single or double mutations, as stated. The flocculation index of the cultures was determined as described in Methods. Data is presented as the mean ± S.E. from 4-5 independent determinations. Statistical significance was determined by unpaired Student's t- test. (*) $p < 0.05$, (**) $p > 0.01$, (***)$p < 0.001$.

Supplementary File 5. Relative specific activity of diverse Ppz1 alleles. A) SDS-PAGE analysis and Coomassie Blue staining of typical recombinant Ppz1 samples upon removal of the GST tag. Std, Molecular Mass standards, WT, wild type Ppz1. The red arrows denote the band corresponding to Ppz1. B) The activity of diverse recombinant Ppz1 variants was determined and compared with that of native Ppz1, which was taken as the unit. The assays were carried out using from 0.5 to 1 µg of recombinant phosphatase preparations. Data is presented as the mean ± S.E. from 5 to 9 determinations from at least five different protein preparations.

Supplementary File 6. Growth curves of *slt2* cells expressing alleles of Ppz1 carrying two amino acid mutations. Strain JC010 (*slt2*) was transformed with the indicated pRS316-based Ppz1 variants and growth was monitored as in Supplementary File 2. For comparison, the absorbance after 20 h of growth is denoted by a blue discontinuous line.

Supplementary File 1. Characterization of selected clones. (+++) strong, (++) medium, (+) weak phenotype. Phenotypic score was based on the capacity of the clones to grow in the presence of increasing concentrations of caffeine. Asterisks denote clones studied in detail in this work.

clone ID	Ident. clones	Nt position	Codon change	AA change	Phenotype	Notes
75 (*)	144	1331	TTC → TCC	Phe → Ser	+++	
		1814	GTT → GCT	Val → Ala		also in #85, 100, 120, 140, and 144
81		1066	AAA → GAA	Lys → Glu	+++	
		1272	TAT → TAC	*none*		
		1942	TTT → CTT	Phe → Leu		
		1951	TTA → CTA	*none*		
		2066	GAA → GGA	Glu → Gly		
		2088	(non coding)	*none*		
83		1672	GAT → AAT	Asp → Asn	++	
		1137	AGA → AGC	Arg → Ser		same nt as in #113, but AA change
85	100, 120, 140	1070	TTC → TCC	Phe → Ser	+++	
		1806	GCT → GCC	*none*		
		1814	GTT → GCT	Val → Ala		as in #75
		1845	GAT → GAC	*none*		
97 (*)	141	1889	GAA → GGA	Glu → Gly	+++	
99 (*)		1282	TTA → GTA	Leu → Val	+	
		2028	CAA → CAG	*none*		
100	85, 120, 140	1070	TTC → TCC	Phe → Ser	+++	
		1806	GCT → GCC	*none*		
		1814	GTT → GCT	Val → Ala		as in #75
		1845	GAT → GAC	*none*		
113		1055	AAC → ACC	Asn → Thr	+++	
		1137	AGA → AGG	*none*		same nt as in #83, no AA change
		1628	CAT → CGT	His → Arg		
		1944	TTT → TTG	Phe → Leu		
		1978	AAG → GAG	Lys → Glu		
115		1788	TTT → TTG	Phe → Leu	+	
		2075	CAG → CCG	Gln → Pro		
116 (*)		1556	ATC → AGC	Ile → Ser	+	
119		1274	GGG → GAG	Gly → Glu	+	
		1572	ATT → ATC	*none*		
		1990	AAG → GAG	Lys → Glu		
120 (*)	85, 100, 140	1070	TTC → TCC	Phe → Ser	+++	
		1806	GCT → GCC	*none*		
		1814	GTT → GCT	Val → Ala		
		1845	GAT → GAC	*none*		
121 (*)	138, 153, 168	1306	TTC → CTC	Phe → Leu	+++	follows single in #141
		1893	TTT → TTG	Phe → Leu		
124 (*)		1724	GAA → GGA	Glu → Gly	+++	also in #139 and #143, no change in nt 1296
125 (*)	126	1504	AAG → CAG	Lys → Gln	++	
		1881	TAT → TAC	*none*		
126	125	1504	AAG → CAG	Lys → Gln	++	
		1881	TAT → TAC	*none*		
129 (*)		1094	GAA → GGA	Glu → Gly	+++	
		1721	AAC → AGC	Asn → Ser		previous to single in #124
131		1478	GAT → AAT	Asp → Asn	++	
		2021	AAT → AGT	Asn → Ser		

132 (*)		1538	ACA → GCA	Thr → Ala	+	
133		1065	AAA → AAC	Lys → Asn	+	
		1224	TTA → TTG	*none*		
		1810	ATG → CTG	Met → Leu		
136		1165	GAA → AAA	Glu → Lys	+	
138	121, 153, 168	1306	TTC → CTC	Phe → Leu	++	
		1893	TTT → TTG	Phe → Leu		
139		1296	ACC → ACT	*none*	+++	also in #124 and #143
		1724	GAA → GGA	Glu → Gly		
140	85, 100, 120	1070	TTC → TCC	Phe → Ser	+++	
		1806	GCT → GCC	*none*		
		1814	GTT → GCT	Val → Ala		as in #75
		1845	GAT → GAC	*none*		
141	97	1889	GAA → GGA	Glu → Gly	+++	
143		1296	ACC → ACT	*none*	+++	also in #124 and #139
		1724	GAA → GGA	Glu → Gly		
144	75	1814	GTT → GCT	Val → Ala	+++	as in #75
		1331	TTC → TCC	Phe → Ser		
145		1924	GAA → CAA	Glu → Gln		
		1955	GAT → GGT	Asp → Gly	+	
		2038	ATG → CTG	Met → Leu		
148		1143	AAA → AAT	*none*	++	
		1144	AAT → GAT	*none*		
		1089	ATT → ATC	*none*		
		1443	GAA → GAG	*none*		
		1763	AAC → AGC	Asn → Ser		
		1830	TAT → TAC	*none*		
		2011	AAA → GAA	Lys → Glu		
149 (*)		1128	GCT → GCC	*none*	+	
		2053	ACA → CCA	Thr → Pro		
151 (*)		1278	GAT → GAC	*none*	++	
		1735	AGT → GGT	Ser → Gly		
		2111	3'-non coding	*none*		
153	121, 138, 168	1306	TTC → CTC	Phe → Leu	+++	
		1893	TTT → TTG	Phe → Leu		
158		1659	TTT → TTA	Phe → Leu	+	
		1731	GGT → GGC	*none*		
		1772	TTG → TCG	Leu → Ser		
167		1135	AGA → GGA	Arg → Gly	+	same AA than #83, different nt
		1148	GTT → GCT	Val → Ala		
		1422	TTT → TTC	*none*		
		1516	ACA → TCA	Thr → Ser		
		1778	AAG → AGG	Lys → Arg		
		1944	TTT → TTC	*none*		same nt than #113, but no AA change
168	121, 138, 153	1306	TTC → CTC	Phe → leu	+	
		1893	TTT → TTG	Phe → Leu		
179		2091	3'-non coding	*none*	+	

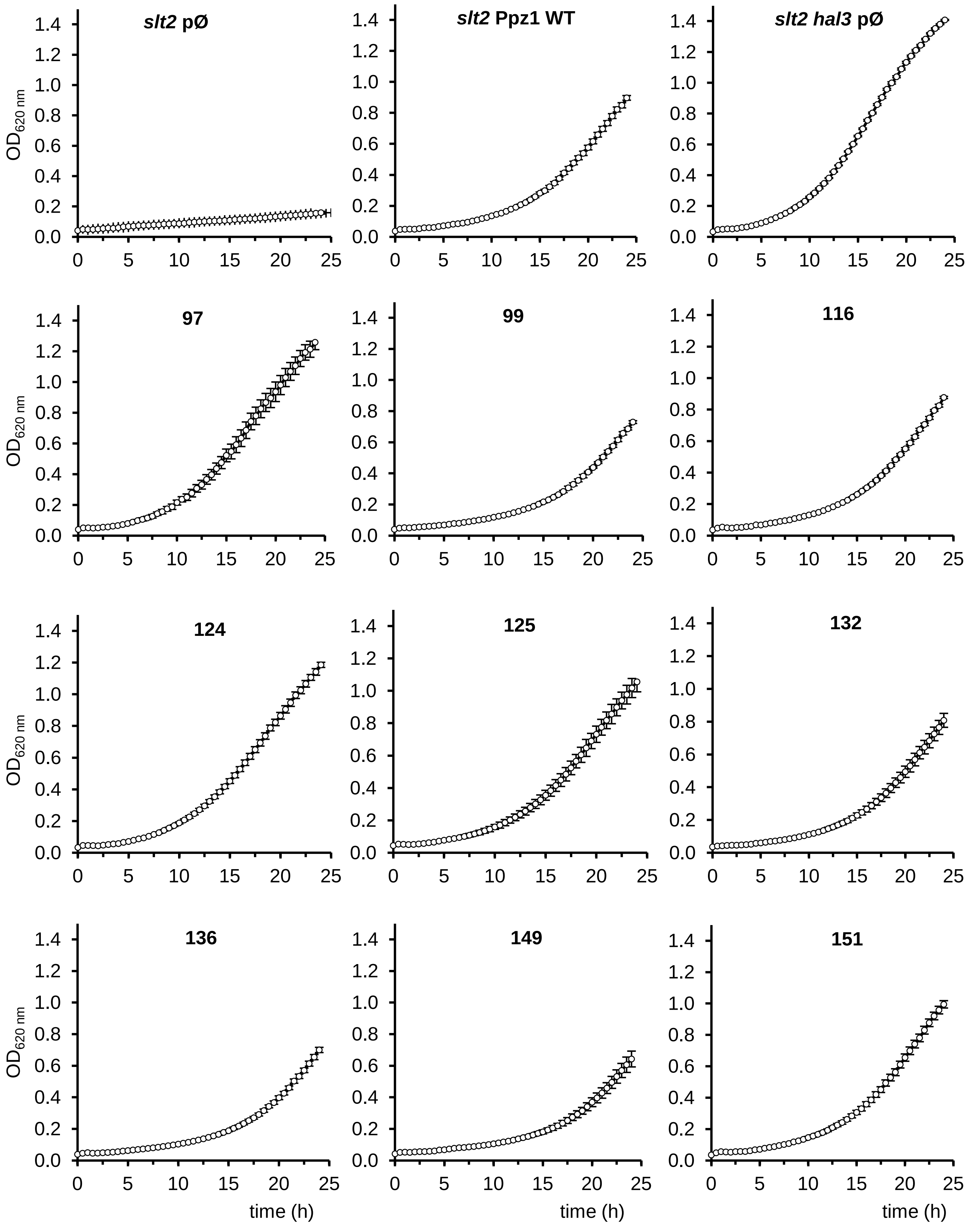

Supplementary File 2

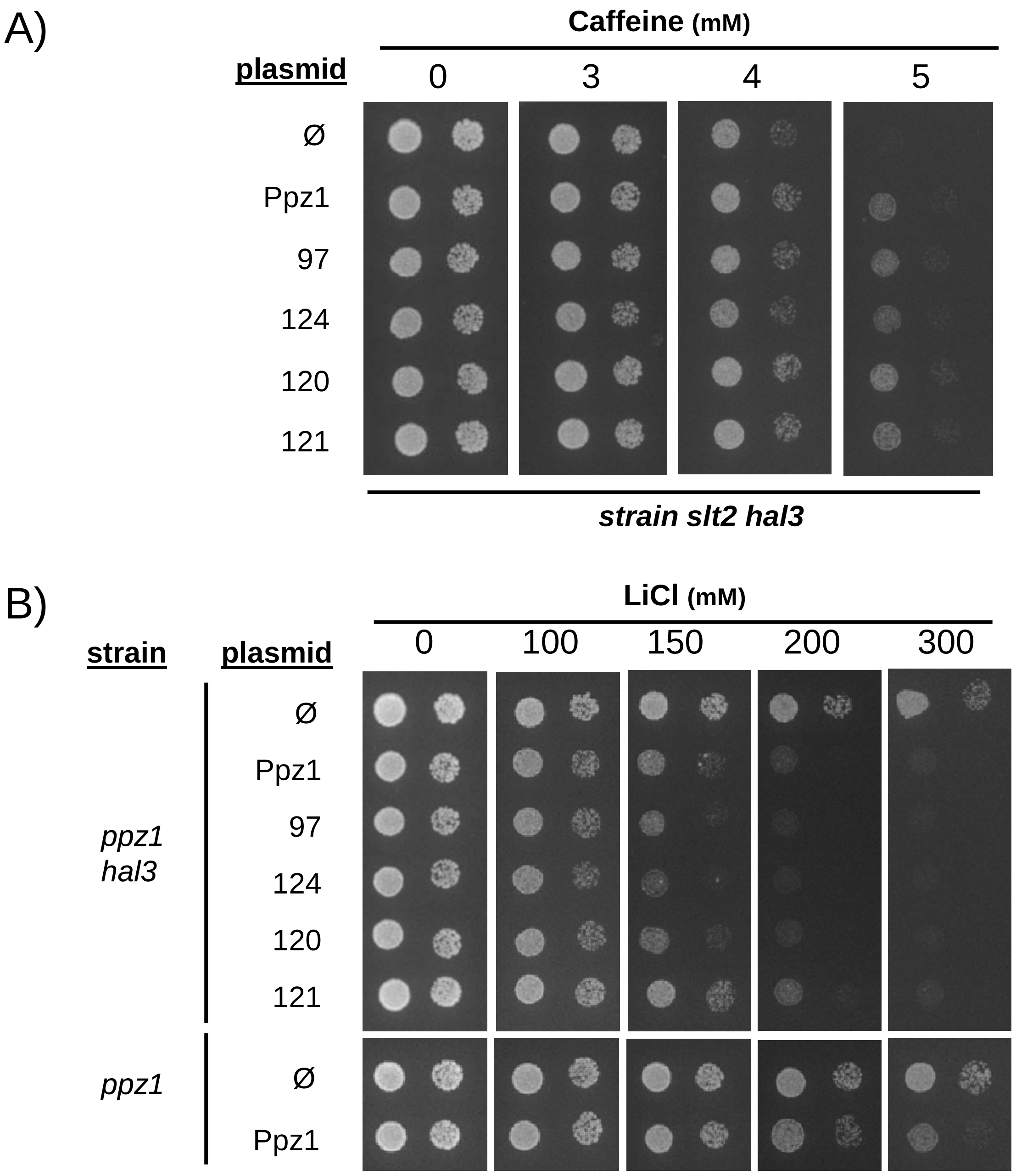

Supplementary File 3

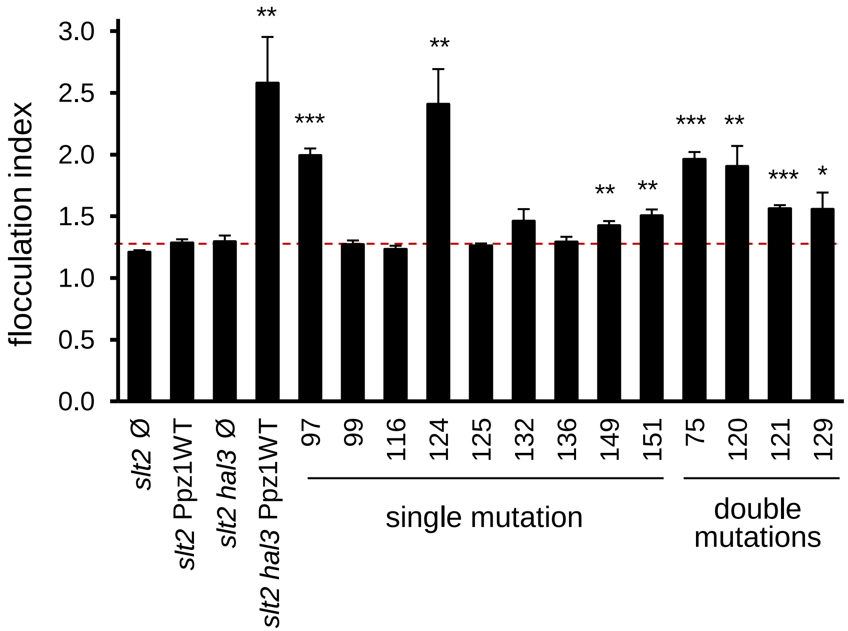

Supplementary File 4

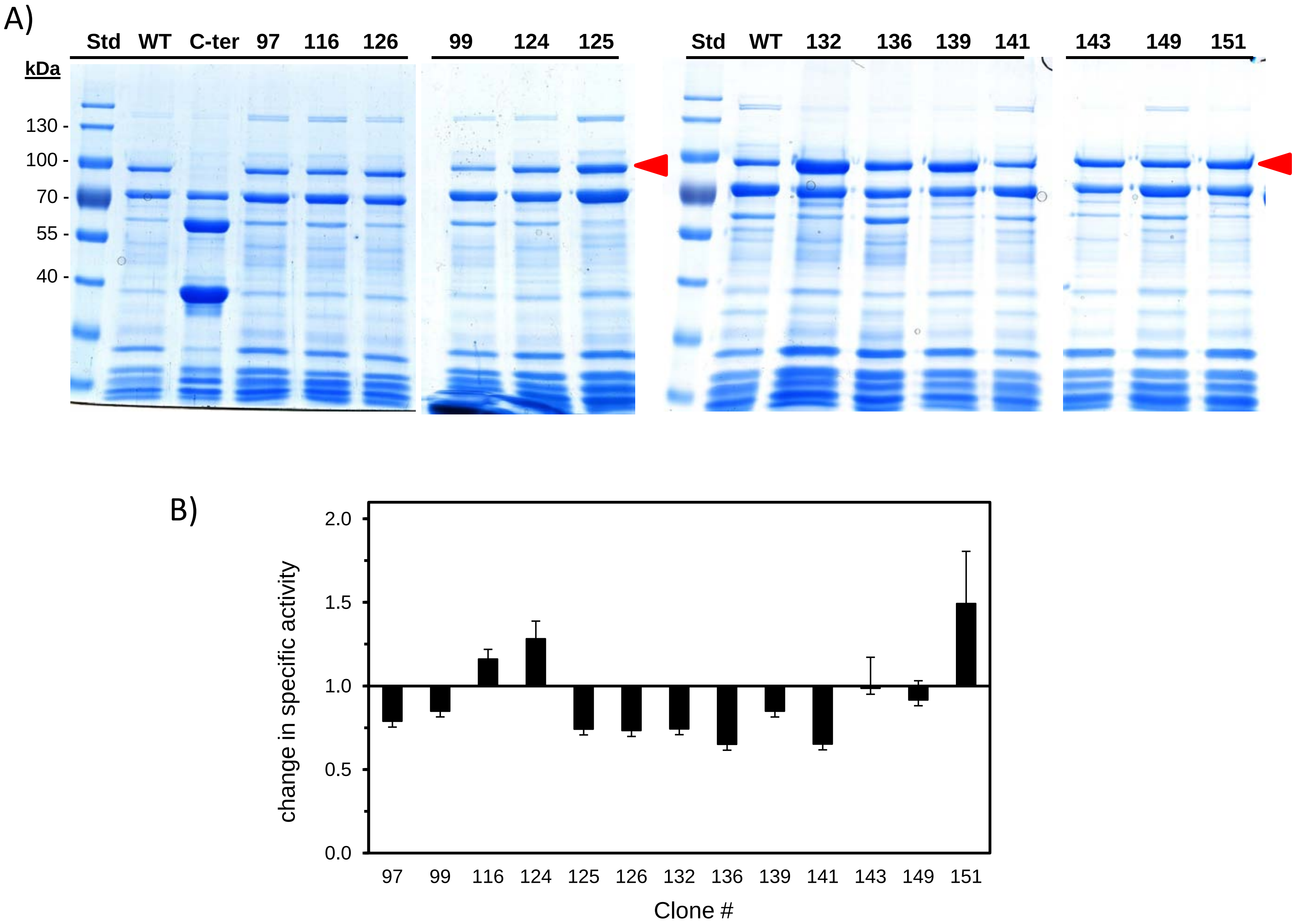

Supplementary File 5

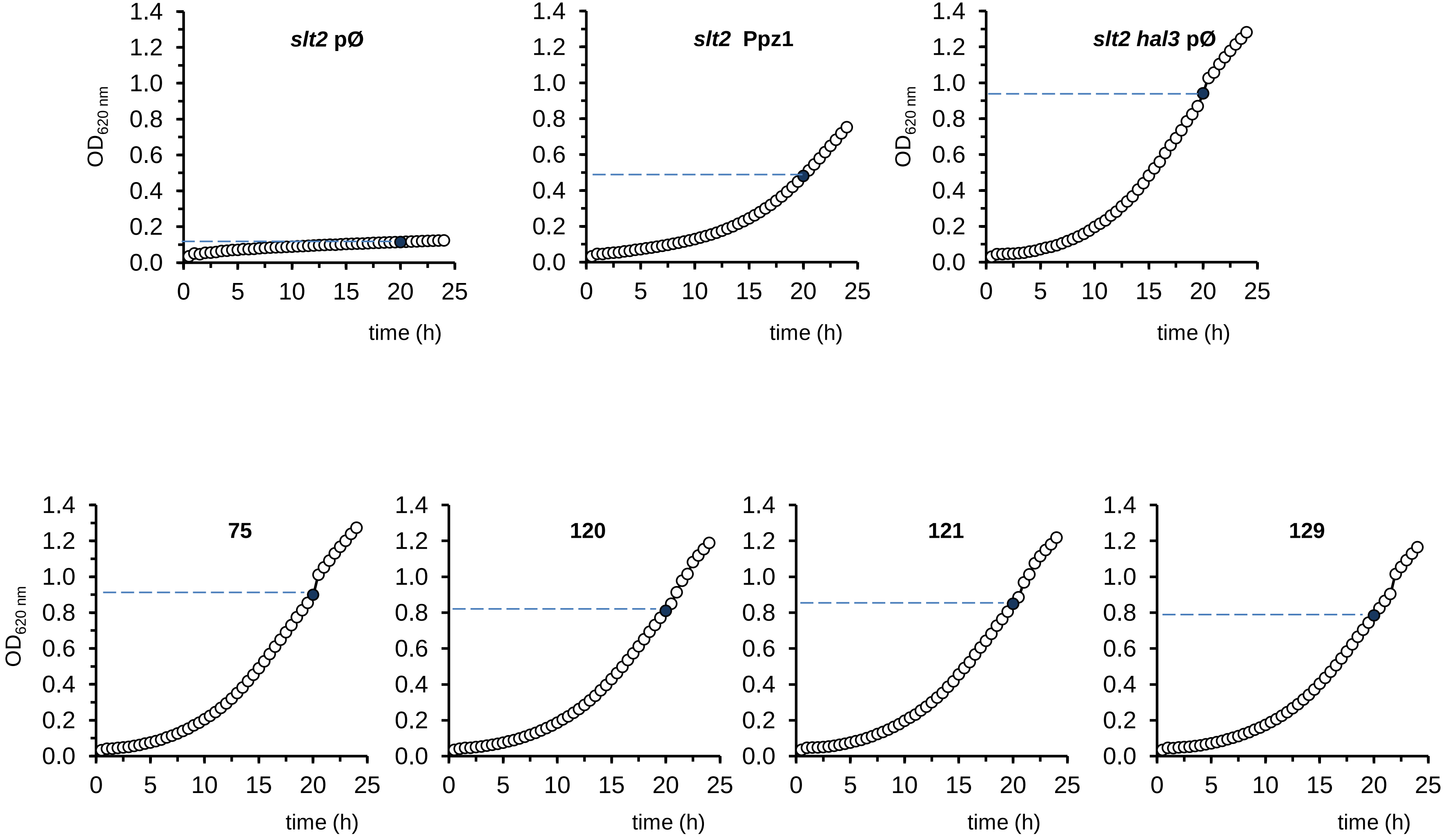

Supplementary File 6
